# A podiatry-led Peripheral Arterial Disease service – observations and early outcomes

**DOI:** 10.1186/1757-1146-3-S1-O10

**Published:** 2010-12-20

**Authors:** Martin Fox, Lisa Smith, Louise Stuart, Helene Gordon, Michelle Proudman

**Affiliations:** 1 NHS Manchester, Manchester, UK

## Introduction

Peripheral Arterial Disease (PAD) is significant issue in the UK. Under diagnosis and management results in avoidable heart attacks, strokes and amputations. Our NHS organisation has commissioned a new Podiatry-led, multidisciplinary, community-based, PAD service. It is focussed on stimulating identification, diagnosis and management, as part of an approach to address poor local cardiovascular disease (CVD) outcomes. We will outline the development and description of the service model and present initial outcomes from it. This will complement the key conference theme of peripheral arterial disease, as presented by Prof. Cliff Shearman.

## Methods

We describe how we used best clinical evidence (SIGN, 2006) as a foundation for defining the clinical content and pathways. The service specification outlined key areas for delivery and progress reporting. We will give an overview of our service development plan, which has focussed on developing proactive initiatives and outcomes in these areas, via broad stakeholder engagement.

## Results

Results on referral patterns, subsequent PAD diagnosis & severity, treatment and patient satisfaction will be presented.

## Discussion

Discussion of the added value of this service within the traditional NHS Podiatry / PAD / CVD service landscape will be presented, along with recommendations for further strengthening the service model.

